# Supplementary figures and images for: Deciphering the microRNA transcriptome of skeletal muscle during porcine development
Source: PeerJ. 2016 Jan 7;4:e1504. doi: 10.7717/peerj.1504 (PMC4715453; doi:10.7717/peerj.1504)

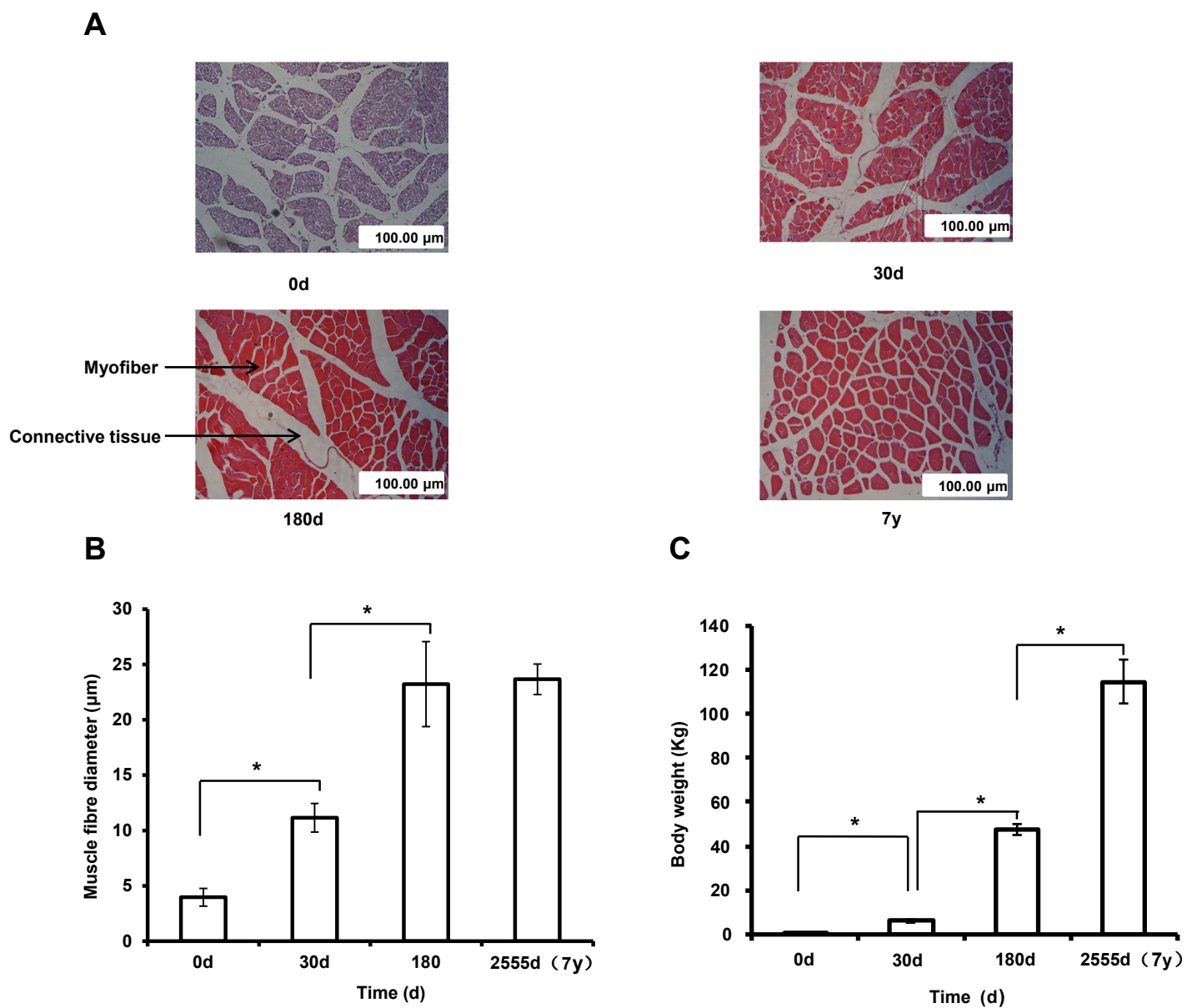

**Figure S1** Muscle fiber diameter and body weight during the postnatal muscle development stage.

Supplement: Figure S1 [file peerj-04-1504-s001.pdf]

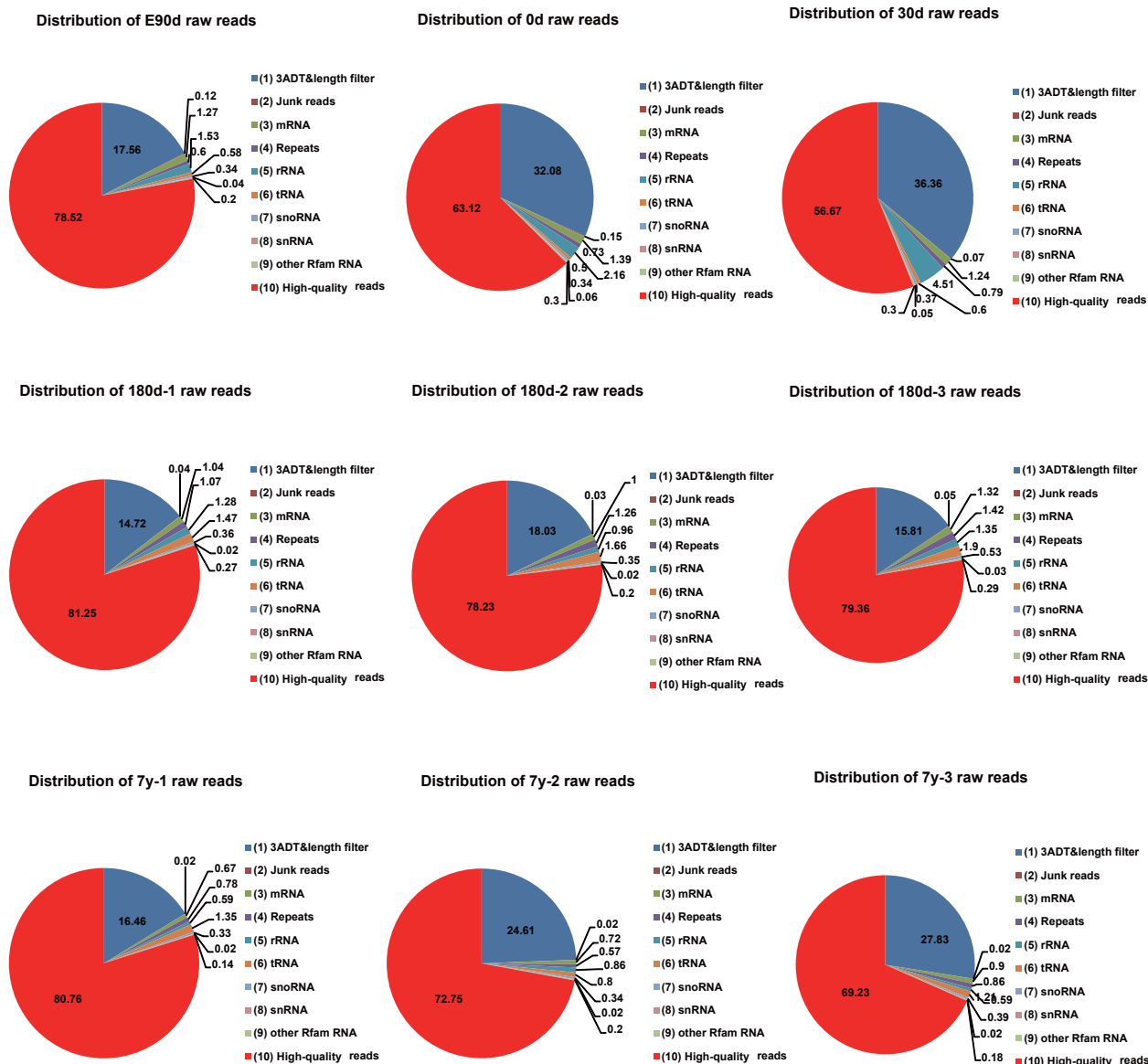

**Figure S2 Distribution of raw reads in each library after applying a series of filters.**

Supplement: Figure S2 [file peerj-04-1504-s002.pdf]

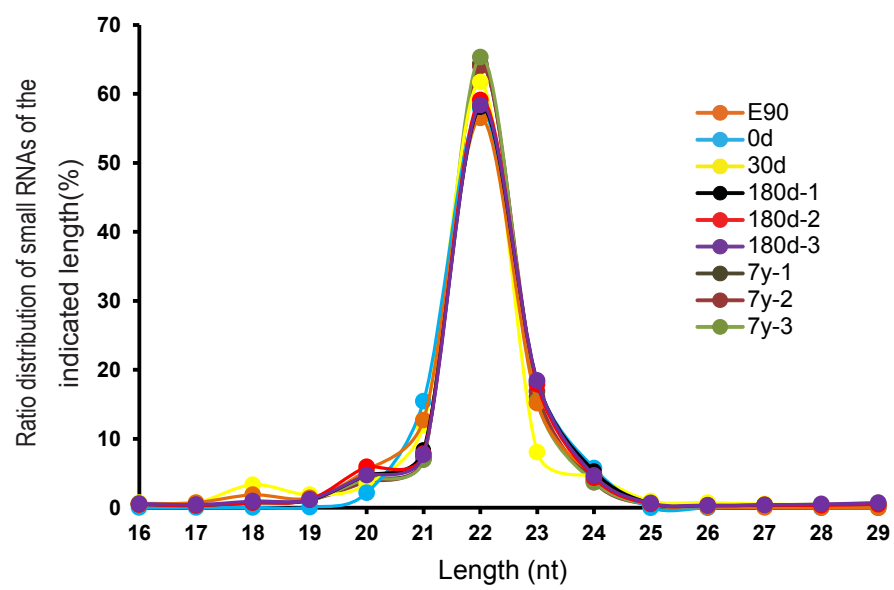

**Figure S3 Length distribution of mappable reads.**

Supplement: Figure S3 [file peerj-04-1504-s003.pdf]

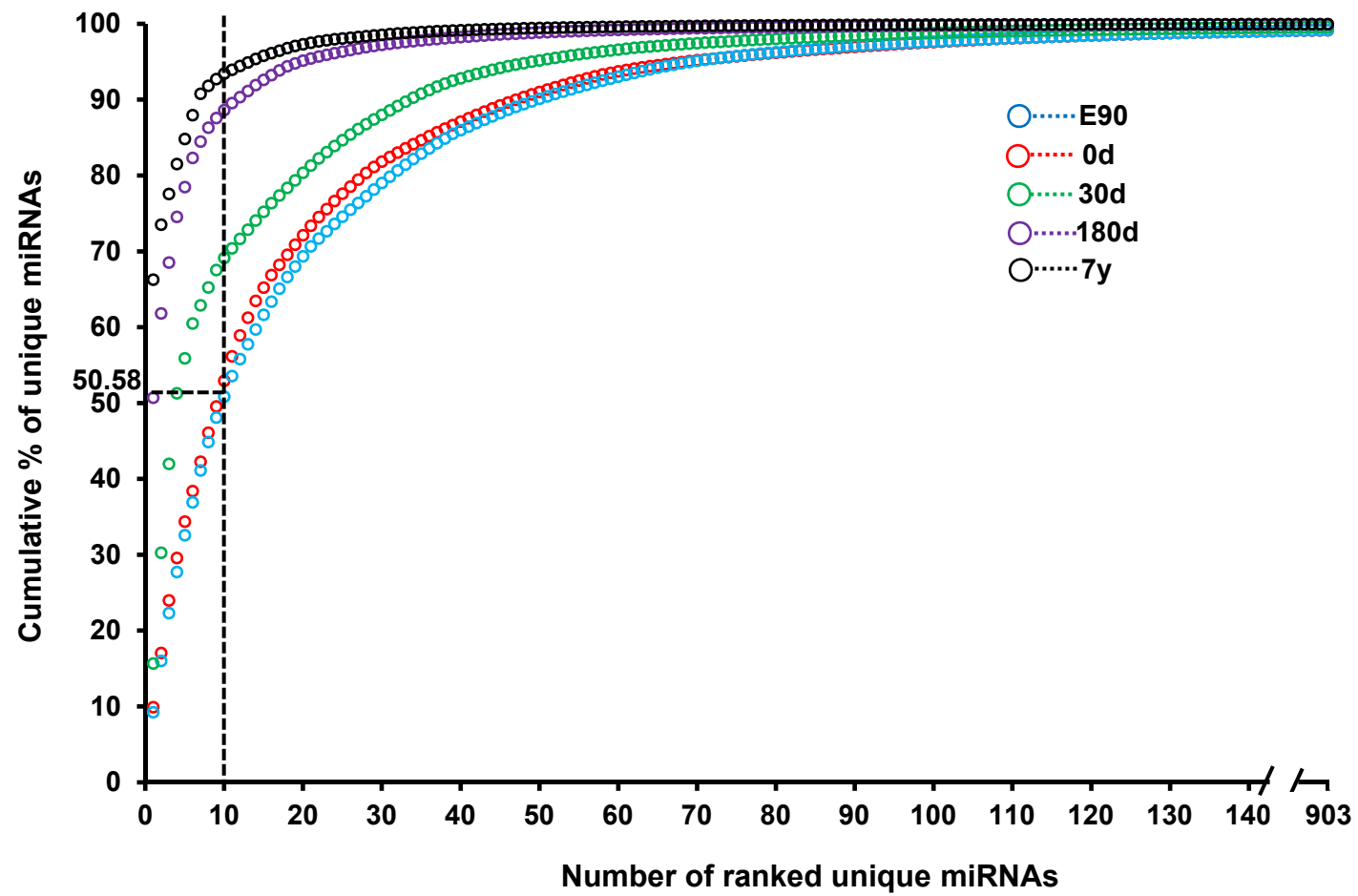

Figure S4 Cumulative distribution of unique miRNAs.

Supplement: Figure S4 [file peerj-04-1504-s004.pdf]
