# Supplementary material for: Deciphering the microRNA transcriptome of skeletal muscle during porcine development
Source: PeerJ. 2016 Jan 7;4:e1504. doi: 10.7717/peerj.1504 (PMC4715453; doi:10.7717/peerj.1504)
Supplement: Table S1 [file peerj-04-1504-s005.docx]

**Table S1** Primer sequences of the q-PCR experiments

| **New Name** | **Primer** | **Primer sequence (5' - 3')** |
| --- | --- | --- |
| miRNA-133a-5p | FW Primer | AGCTGGTAAAATGGAACCAAAT |
| miR-181a-1-3 | FW Primer | AACATTCAACGCTGTCGGTGAGTT |
| miR-499-5p | FW Primer | TTAAGACTTGCAGTGATGTTT |
| miR-320-3p | FW Primer | AAAAGCTGGGTTGAGAGGGCGAA |
| miR-24-1-3p | FW Primer | GTGCCTACTGAGCTGAAACACAGT |
| miR-214-3p | FW Primer | ACAGCAGGCACAGACAGGCAG |
| let-7g-5p | FW Primer | TGAGGTAGTAGTTTGTACAGTT |
| miR-23a-3p | FW Primer | ATCACATTGCCAGGGATTTCC |
| miR-10b-3p | FW Primer | TACCCTGTAGAACCGAATTTGT |
| U6 snRNA | FW Primer | TTATGGGTCCTAGCCTGAC |
|  | RW Primer | CACTATTGCGGGTCTGC |
| 5S rRNA | FW Primer | GCCCGATCTCGTCTGATCT |
|  | RW Primer | AGCCTACAGCACCCGGTATT |
| 18S rRNA | FW Primer | TTTCGCTCTGGTCCGTCTTG |
|  | RW Primer | TTCGGAACTGAGGCCATGAT |
